# Anxiety and Insomnia Among Urban Slum Dwellers in Bangladesh: The Role of COVID-19 and Its Associated Factors

**DOI:** 10.3389/fpsyt.2021.769048

**Published:** 2021-12-03

**Authors:** Kamrun Nahar Koly, Mosammat Ivylata Khanam, Md. Saiful Islam, Shehrin Shaila Mahmood, Syed Manzoor Ahmed Hanifi, Daniel D. Reidpath, Fatema Khatun, Sabrina Rasheed

**Affiliations:** Health System and Population Studies Division, International Centre for Diarrhoeal Disease Research, Bangladesh (icddr,b), Dhaka, Bangladesh

**Keywords:** public mental health, marginalized communities, slum dwellers, COVID-19, pandemic, emergency

## Abstract

**Background:** Although mental health is an important part of health and wellbeing, very little is known about the impact of the COVID-19 pandemic on the mental health of marginalized communities like urban slum dwellers. Our study estimated the prevalence of generalized anxiety disorder and insomnia among the residents of the informal settlements of Dhaka, Bangladesh, during the COVID-19 pandemic.

**Methods:** A cross-sectional phone-based survey was conducted from October to November 2020 among adult residents of five informal settlements of Dhaka city randomly chosen from an existing Urban Health and Demographic Surveillance Systems (UHDSS) run by icddr,b. Data on Generalized Anxiety Disorder-7 (GAD-7) and Insomnia Severity Index (ISI) were collected. A multinomial logistic regression was performed to assess the associated factors of anxiety and insomnia.

**Results:** Of the total 586 participants, the prevalence of mild to severe anxiety and insomnia were 53% and 43%, respectively. As per the multinomial regression analysis, participants with mild anxiety were significantly more likely to be older (>50 years) and afraid of COVID-19 infection. Likewise, participants with moderate/severe anxiety were significantly more likely to share less household facilities (e.g., toilet, kitchen, water) (OR: 2.23; 95% CI: 1.31–3.79), to have difficulties in food availability (OR: 2.76; 95% CI: 1.10–6.93), to be afraid of self (OR: 5.27; 95% CI: 2.82–9.88), and to worry about the family members (OR: 2.26; 95% CI: 1.23–4.17) getting infected. Participants with mild insomnia were significantly more likely to share fewer household facilities and be afraid of being infected with COVID-19 infection. Moreover, participants with moderate/severe insomnia were significantly more likely to be female (OR: 1.90; 95% CI: 1.02–3.56), to receive food aid (OR: 0.50; 95% CI: 0.29–0.88), to be afraid of self (OR: 3.85; 95% CI: 1.81–8.19), and to worry about someone like friends or neighbors (OR: 2.45; 95% CI: 1.07–5.58) getting infected with COVID-19.

**Conclusions:** We found elevated prevalence of both anxiety and insomnia among the urban poor of Bangladesh in the context of COVID-19. This indicates the importance of integrating mental health in the mitigation and recovery efforts related to similar crises for the urban poor in the future.

## Background

Globally, the urban population has increased rapidly from 751 million in 1950 to 4.2 billion in 2018, and it is projected to increase by 68% within 2050 (1). Currently, worldwide more than 900 million people reside in the urban slums, and around 7 million live in ~3,394 slums of Dhaka, Bangladesh (2). Some of the challenges of living in urban slums include poor infrastructure, overcrowding, inadequate water supply, improper waste management, and less access to health services (3–7). The amenities available for daily living such as water and kitchen and toilet facilities are often shared by residents of the slums (8). The lives of urban working poor people were severely affected by the COVID-19 pandemic as they lost income and had to deal with the rising costs of living (8). During the COVID-19 pandemic, when movement restrictions, frequent hand washing, and maintaining social distance were promoted as preventive measures, compliance with these recommendations was particularly challenging for the residents of urban slums due to the environment they live in (9–11). In addition, several researchers have reported the poor mental health status of the slum residents due to their vulnerable socioeconomic status and the poor infrastructure of the slums in Bangladesh (12–14). The crowded living and inadequate infrastructure meant that this population was at higher risk of transmitting the disease both inside and outside their area of residence (8, 9, 15–17). The high risk of disease transmission among the residents of urban slums was likely to contribute to the high rates of COVID-19 infection among Dhaka city residents (18).

Researchers in many low- and middle-income countries (LMICs) have reported that disease outbreaks such as Ebola and influenza have led to a rise in unemployment, malnutrition, gender violence, and reduced access to healthcare services (8, 19, 20). During previous epidemics, researchers have also reported the increased of anxiety, stress, sleep, and appetite disorders (8, 21, 22). Similarly, during the current pandemic, researchers have reported rise in unemployment, food insecurity (rural and urban areas), violence, and reduction in healthcare seeking (8, 23, 24). Although the abovementioned stressors along with fear of contracting COVID-19 and stigma related to the pandemic are likely to manifest as stress and anxiety among the urban poor, very few studies have investigated the mental health implications of the pandemic for the residents of urban slums.

During the COVID-19 pandemic, there are a few studies that focused on the mental health of urban poor in Bangladesh (25–27). Most of these have investigated depression and stress and used convenient sampling procedures (25, 26). A previous study indicated the presence of anxiety and sleeping problems among slum dwellers during the pandemic (24). However, the study was conducted in a small sample and was exploratory in nature. Therefore, this study focused on the quantitative assessment of anxiety and insomnia using standardized tools among a representative sample of residents of urban slums in Dhaka city. It was hypothesized that the prevalence of anxiety and insomnia would be higher during the pandemic compared to the previous rates among the residents of urban slums in Dhaka city. The samples were drawn from an existing surveillance system of urban slum residents of Dhaka. Therefore, these study findings will help to inform future policies and programs related to the urban poor.

## Materials and Methods

### Study Design and Setting

A cross-sectional survey was conducted among slum dwellers in Dhaka city, Bangladesh, under the Urban Health and Demographic Surveillance System (UHDSS) of icddr,b. It is a large surveillance system of icddr,b since 2015 covering 31,577 households and 121,912 individuals in five slums of the Korail, Mirpur, Shampur, Dholpur, and Gazipur areas of Dhaka North and South city corporations. Households were selected randomly with a probability proportional to size (PPS) so that the population represents the urban slums in general. From each household, one adult (≥ 18 years of age) with phone number registered with UHDSS was selected for the study interview ensuring that the male–female ratio remained similar. The residents who had migrated permanently out of the slum were excluded from the study.

### Sampling

As no similar studies among slum dwellers during the pandemic were available at the designing of the study, we assumed the prevalence of anxiety to be 50%. The minimum sample size (384) was estimated with an assumption of 50% poor mental health conditions with a 5% precision. Finally, a total of (384 × 1.5) 578 households were calculated with the design effect (1.5), and taking 20% non-response into consideration, ~695 households were estimated. Afterward, we determined the number of the respondents from each slum by using PPS methodology. Initially, a total of 1,519 adult slum residents (one from each household) were approached to take part in the survey. Finally, 586 participated and completed the survey, whereas 65 respondents refused to participate and 902 residents were unable to reach by phone, generating a non-response rate of 61.4%.

### Data Collection Procedure

To avoid moving around and face-to-face contact during the government lockdown (movement and social restriction), a mobile phone-based survey was conducted. Trained research assistants who were already working within the selected communities and obtained data through phone calls after getting verbal consent by using a Bangla pretested questionnaire from the respondents. The study protocol was reviewed and approved by the Institutional Review Board (IRB) of icddr,b.

### Data Collection Tools

A semi-structured questionnaire was used for data collection that included sections on socio-demographics including questions on age, sex, years of schooling, occupation, and income. Other sections included COVID-19 impact on household characteristics, food environment, and emotional wellbeing characteristics. Under the section household characteristics, we have assessed crowding index (number of people per room) and shared facilities (sharing of kitchen, toilet, and water supply). We collected questions about food availability, accessibility, and food aid under the section of food environment. Emotional wellbeing had some variables such as fear of contracting the COVID-19 infection for self, family members, and other close network; in addition, this section also measured anxiety and insomnia using two psychometric scales that have been used previously in the Bangladeshi population: the Generalized Anxiety Disorder-7 (GAD-7) scale and the Insomnia Severity Index (ISI) (28, 29).

#### Generalized Anxiety Disorder

The Generalized Anxiety Disorder (GAD-7) includes seven questions about symptoms of anxiety experienced over the last 2 weeks. Each question is scored on a four-point Likert scale (from “0 = not at all” to “3 = almost every day”). The total score ranges from 0 to 21 and is obtained by summing the raw scores of the seven items. A higher score is indicative of greater anxiety. In the present study, the following GAD-7 cutoffs were considered for assessing anxiety: > 15 for “severe anxiety”; 10–14 for “moderate anxiety”; 5–9 for “mild anxiety”; and < 5 considered was “minimal anxiety” (28). The Cronbach's alpha for GAD-7 in the present study was 0.94, indicating excellent reliability.

#### Insomnia Severity Index

The Insomnia Severity Index (ISI) scale consists of seven items related to symptoms of insomnia over the past 2 weeks scored on a five-point Likert scale (e.g., “0 = Not at all” to “4 = Nearly every day”). The total score is obtained by summing the item scores. Individual scores range from 0 to 28, with higher scores reflecting more severe insomnia. The following ISI cutoffs were used for assessing insomnia; 22–28 for “severe insomnia”; 15–21 for “moderate insomnia”; and 8–14 for “mild” or “subthreshold insomnia”—and < 8 was considered as “minimal insomnia” (29). The Cronbach's alpha for the ISI in the present study was 0.80.

### Data Analysis

The descriptive statistics were computed for each variable. The bivariate analysis (i.e., Chi-square tests, Fisher's exact tests) were computed to assess the association between independent variables and outcome variables (i.e., anxiety and insomnia). Multinomial logistic regression analysis was performed involving all examined variables together to determine the associated factors of anxiety and insomnia categories (mild and moderate/severe). During regression analysis, “minimal” categories were kept as reference. Before performing the multinomial logistic regression models, multi-collinearity was checked. The multi-collinearity was absent as all values of the tolerance and variance inflation factor (VIF) were > 0.1 and < 2, respectively (30). All the studied variables were based on the previous literature which were included in the multinomial logistic regression models. Thus, it is expected that all the potential confounders have been adjusted. A *p* < 0.05 was considered as a statistically significant association. SPSS software (version 25) was used to analyze data.

## Results

### General Characteristics of the Participants

Out of the 586 study participants, 50.9% were males; 48.6% were of 31–50 years old; 50.9% had 5–10 years of schooling; and 35.2% were housewives or students followed by 30.2% unemployed or daily wager. About 93.7% of respondents reported that COVID-19 had adversely affected their household income (Table 1).

**Table 1:** Characteristics of the participants.

**Variable**	**Category**	**% of the sample** **(*n* = 586)**
Age	31–50 years	48.6
	>50 years	10.6
	18–30 years	40.8
Sex	Female	49.1
	Male	50.9
Years of schooling	<5 years	43.2
	5–10 years	50.9
	>10 years	6.0
Occupation	Unemployed/day laborer	30.2
	Service	15.4
	Business/self-employed/garments worker	19.3
	Student/housewives/other	35.2
Household income affected by COVID-19	Yes	93.7
	No	6.3
Crowding index	Over-crowded	73.9
	Normal	26.1
Shared facilities	0 to 1	24.2
	2 or more	75.8
Food availability for the household	Difficulties	89.2
	No difficulties	10.8
Food purchase	Bought less	87.4
	More bought/same as before	12.6
Any food aid received	No	49.7
	Yes	50.3
Afraid of being infected by COVID-19	Yes	33.3
	No	66.7
Worried about family members to be infected	Yes	33.8
	No	66.2
Someone like friends or neighbors get infected	Yes	8.0
	No	92.0

### Prevalence of Anxiety and Insomnia Among Urban Slum Residents

**Figure 1:**
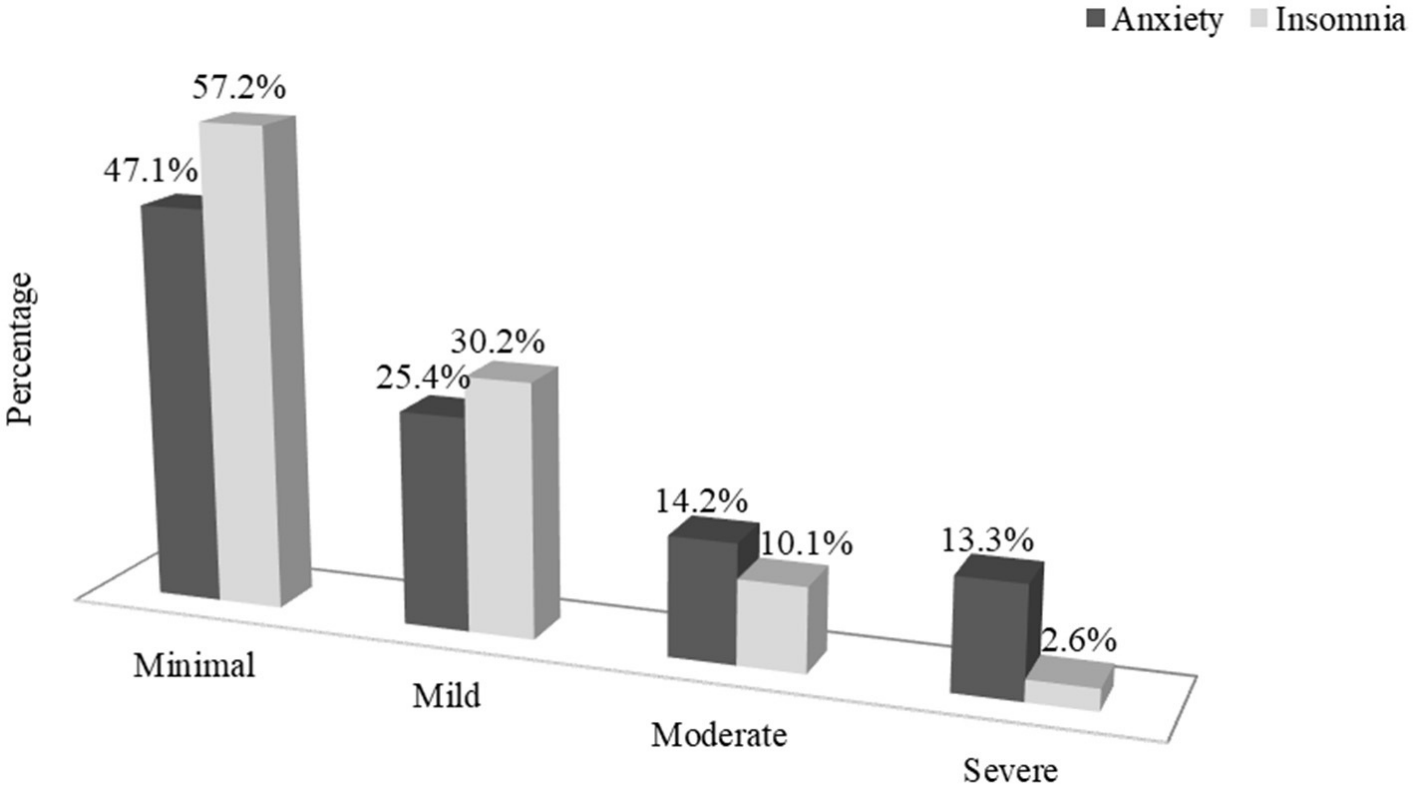
Estimation of anxiety and insomnia among the urban slum residents.

Among the 586 study participants, 53.0% had mild to severe anxiety, and 43.0% had mild to severe insomnia (Figure 1). About a quarter of respondents had mild anxiety symptoms (25.4%), and a little under one-third had mild insomnia symptoms (30.2%). About 14.2% of respondents experienced moderate anxiety, and 10.1% had moderate insomnia. Severe anxiety was observed in about 13.3% of people and 2.6% experienced severe insomnia.

**Figure 2:**
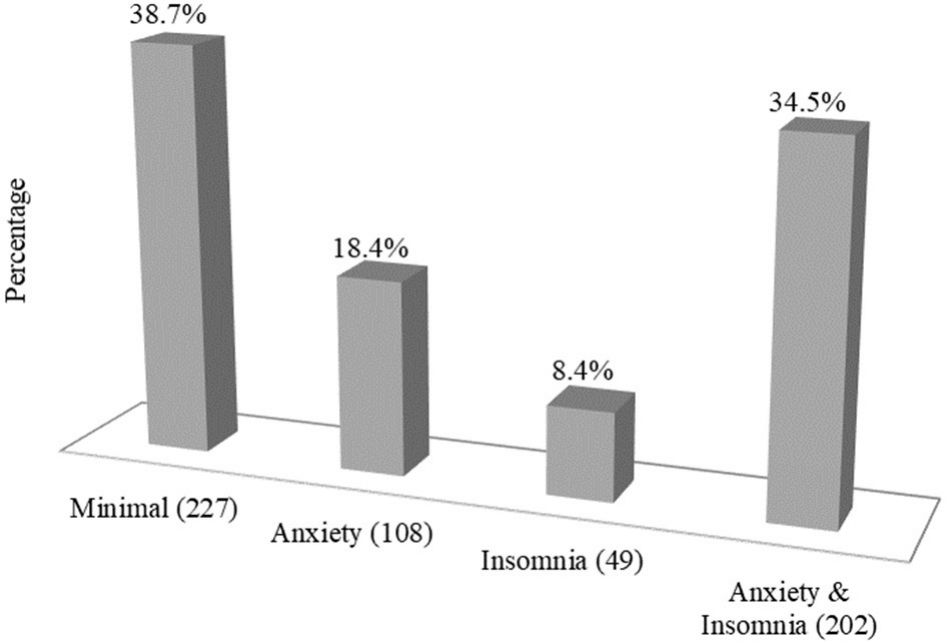
Overall prevalence of anxiety and insomnia among the urban slum residents.

Figure 2 represents the participants' symptoms of anxiety and insomnia during the COVID-19 pandemic. About 61.3% of the slum residents experienced either anxiety or insomnia or both, 34.5% had both anxiety and insomnia symptoms, 18.4% experienced only anxiety, and 8.4% experienced only insomnia.

### Distribution of Anxiety and Insomnia

The distributions of minimal, mild, and moderate/severe anxiety and insomnia are presented in Tables 2, 3. During the Chi-square test, we found that anxiety was significantly associated with shared facilities, food availability, food purchasing ability, being afraid of being infected by COVID-19, and being worried about family members being infected (Table 2). On the other hand, insomnia was significantly associated with sex, shared facilities, food purchasing ability, being afraid of being infected by COVID-19, being worried about family members being infected, and having someone like friends or neighbors getting infected (Table 3).

**Table 2:** Associations between independent variables and levels of anxiety.

		**Anxiety**			**Test of association**		
		**Minimal** ** (*n* = 276)**	**Mild** ** (*n* = 149)**	**Moderate/** **severe** ** (*n* = 161)**			
**Variable**	**Category**	***n* (%)**	***n* (%)**	***n* (%)**	**Total *n***	**χ^2^**	***p*-value**
Age	31–50 years	134 (47)	68 (23.9)	83 (29.1)	285	4.26	0.372
	>50 years	27 (43.5)	22 (35.5)	13 (21)	62		
	18–30 years	115 (48.1)	59 (24.7)	65 (27.2)	239		
Sex	Female	124 (43.1)	79 (27.4)	85 (29.5)	288	3.72	0.156
	Male	152 (51)	70 (23.5)	76 (25.5)	298		
Years of schooling	<5 years	117 (46.2)	68 (26.9)	68 (26.9)	253	1.14	0.887
	5–10 years	142 (47.7)	71 (23.8)	85 (28.5)	298		
	>10 years	17 (48.6)	10 (28.6)	8 (22.9)	35		
Occupation	Unemployed/day laborer	81 (45.8)	50 (28.2)	46 (26)	177	7.82	0.251
	Service	37 (41.1)	22 (24.4)	31 (34.4)	90		
	Business/self-employed/garments worker	62 (54.9)	20 (17.7)	31 (27.4)	113		
	Student/housewives/other	96 (46.6)	57 (27.7)	53 (25.7)	206		
Household income affected by COVID-19	Yes	253 (46.1)	141 (25.7)	155 (28.2)	549	3.95	0.139
	No	23 (62.2)	8 (21.6)	6 (16.2)	37		
Crowding index	Overcrowded	213 (49.2)	109 (25.2)	111 (25.6)	433	3.63	0.163
	Normal	63 (41.2)	40 (26.1)	50 (32.7)	153		
Shared facilities	0 to 1	54 (38)	39 (27.5)	49 (34.5)	142	6.95	0.031
	2 or more	222 (50)	110 (24.8)	112 (25.2)	444		
Food availability for the household	Difficulties	235 (44.9)	135 (25.8)	153 (29.3)	523	10.74	0.005
	No difficulties	41 (65.1)	14 (22.2)	8 (12.7)	63		
Food purchase	bought less	230 (44.9)	134 (26.2)	148 (28.9)	512	7.99	0.018
	More bought/same as before	46 (62.2)	15 (20.3)	13 (17.6)	74		
Any food aid received	No	150 (51.5)	69 (23.7)	72 (24.7)	291	4.67	0.097
	Yes	126 (42.7)	80 (27.1)	89 (30.2)	295		
Afraid of being infected by COVID-19	Yes	42 (21.5)	57 (29.2)	96 (49.2)	195	92.55	<0.001
	No	234 (59.8)	92 (23.5)	65 (16.6)	391		
Worried about family members to be infected	Yes	50 (25.3)	58 (29.3)	90 (45.5)	198	67.25	<0.001
	No	226 (58.2)	91 (23.5)	71 (18.3)	388		
Someone like friends or neighbors get infected	Yes	16 (34)	17 (36.2)	14 (29.8)	47	4.27	0.118
	No	260 (48.2)	132 (24.5)	147 (27.3)	539		

**Table 3:** Associations between independent variables and levels of insomnia.

		**Insomnia**			**Test of association**		
		**Minimal** **(*n* = 335)**	**Mild** **(*n* = 177)**	**Moderate/** **severe** **(*n* = 74)**			
**Variable**	**Category**	***n* (%)**	***n* (%)**	***n* (%)**	**Total *n***	**χ^2^**	***p*-value**
Age	31–50 years	156 (54.7)	89 (31.2)	40 (14)	285	3.19	0.526
	>50 years	41 (66.1)	16 (25.8)	5 (8.1)	62		
	18–30 years	138 (57.7)	72 (30.1)	29 (12.1)	239		
Sex	Female	152 (52.8)	91 (31.6)	45 (15.6)	288	6.30	0.043
	Male	183 (61.4)	86 (28.9)	29 (9.7)	298		
Years of schooling	<5 years	155 (61.3)	63 (24.9)	35 (13.8)	253	7.353^†^	0.115
	5–10 years	158 (53)	105 (35.2)	35 (11.7)	298		
	>10 years	22 (62.9)	9 (25.7)	4 (11.4)	35		
Occupation	Unemployed/day laborer	102 (57.6)	54 (30.5)	21 (11.9)	177	5.70	0.458
	Service	47 (52.2)	27 (30)	16 (17.8)	90		
	Business/self-employed/garments worker	73 (64.6)	30 (26.5)	10 (8.8)	113		
	Student/housewives/other	113 (54.9)	66 (32)	27 (13.1)	206		
Household income affected by COVID-19	Yes	308 (56.1)	169 (30.8)	72 (13.1)	549	3.92^†^	0.139
	No	27 (73)	8 (21.6)	2 (5.4)	37		
Crowding index	Overcrowded	247 (57)	131 (30.3)	55 (12.7)	433	0.01	0.994
	Normal	88 (57.5)	46 (30.1)	19 (12.4)	153		
Shared facilities	0–1	65 (45.8)	58 (40.8)	19 (13.4)	142	11.36	0.003
	2 or more	270 (60.8)	119 (26.8)	55 (12.4)	444		
Food availability for the household	Difficulties	293 (56)	163 (31.2)	67 (12.8)	523	2.73	0.255
	No difficulties	42 (66.7)	14 (22.2)	7 (11.1)	63		
Food purchase	Bought less	282 (55.1)	162 (31.6)	68 (13.3)	512	7.23	0.027
	More bought/same as before	53 (71.6)	15 (20.3)	6 (8.1)	74		
Any food aid received	No	174 (59.8)	90 (30.9)	27 (9.3)	291	5.93	0.051
	Yes	161 (54.6)	87 (29.5)	47 (15.9)	295		
Afraid of being infected by COVID-19	Yes	67 (34.4)	87 (44.6)	41 (21)	195	63.01	<0.001
	No	268 (68.5)	90 (23)	33 (8.4)	391		
Worried about family members to be infected	Yes	80 (40.4)	80 (40.4)	38 (19.2)	198	35.20	<0.001
	No	255 (65.7)	97 (25)	36 (9.3)	388		
Someone like friends or neighbors get infected	Yes	23 (48.9)	12 (25.5)	12 (25.5)	47	7.71	0.021
	No	312 (57.9)	165 (30.6)	62 (11.5)	539		

† *Fisher's exact test*.

### Multinomial Logistic Regression Analysis

A multinomial logistic regression evaluated the associated factors of anxiety and insomnia categories (mild and moderate/severe). The reference group was minimal anxiety and insomnia, respectively. Participants with mild anxiety were significantly more likely to be of older age (> 50 years) (OR: 2.11; 95% CI: 1.00–4.42, *p* < 0.05) and to be afraid of being infected by COVID-19 (OR: 2.65; 95% CI: 1.41–4.98, *p* < 0.01; Table 4). Likewise, participants with moderate/severe anxiety were significantly more likely to share fewer household facilities (OR: 2.23; 95% CI: 1.31–3.79, *p* < 0.01), to have difficulties in food availability (OR: 2.76; 95% CI: 1.10–6.93, *p* < 0.05), to be afraid of being infected by COVID-19 (OR: 5.27; 95% CI: 2.82–9.88, *p* < 0.001), and to be worried about family members to be infected (OR: 2.26; 95% CI: 1.23–4.17, *p* < 0.01).

**Table 4:** Multinomial logistic regression differentiating minimal anxiety (*n* = 276) from mild and moderate/severe anxiety.

**Variable**	**Category**	**Mild anxiety (*****n*** **= 149)**			**Moderate/severe anxiety (*****n*** **= 161)**		
		***B***	**Wald χ^2^ statistic**	**OR (95% CI)**	***B***	**Wald χ^2^ statistic**	**OR (95% CI)**
Age	31–50 years	0.05	0.04	1.05 (0.65–1.69)	0.14	0.30	1.15 (0.70–1.87)
	>50 years	0.74	3.87	2.11 (1.00–4.42)^*^	0.23	0.28	1.26 (0.54–2.94)
Sex	Female	0.22	0.79	1.25 (0.76–2.05)	0.32	1.44	1.37 (0.82–2.29)
Years of schooling	<5 years	0.07	0.02	1.08 (0.41–2.80)	0.76	1.88	1.08 (0.41–2.80)
	5–10 years	−0.12	0.07	0.89 (0.36–2.21)	0.52	0.97	0.89 (0.36–2.21)
Occupation	Unemployed/day laborer	0.19	0.41	1.20 (0.68–2.13)	0.33	1.15	1.40 (0.76–2.56)
	Service	0.22	0.37	1.25 (0.61–2.56)	0.71	3.59	2.04 (0.98–4.25)
	Business/self-employed/garments worker	−0.41	1.29	0.67 (0.33–1.34)	0.32	0.80	1.37 (0.69–2.75)
Household income affected by COVID-19	Yes	0.46	0.86	1.58 (0.60–4.15)	0.84	2.23	2.31 (0.77–6.94)
Crowding index	Overcrowded	−0.20	0.59	0.82 (0.49–1.37)	−0.35	1.68	0.70 (0.41–1.20)
Shared facilities	0 to 1	0.47	3.18	1.60 (0.95–2.69)	0.80	8.72	2.23 (1.31–3.79)^**^
Food availability for the household	Difficulties	0.24	0.40	1.27 (0.61–2.63)	1.02	4.70	2.76 (1.10–6.93)^*^
Food purchase	Bought less	0.71	3.30	2.04 (0.95–4.40)	0.57	1.80	1.77 (0.77–4.08)
Any food aid received	No	−0.32	2.23	0.72 (0.47–1.11)	−0.43	3.59	0.65 (0.41–1.02)
Afraid of being infected by COVID-19	Yes	0.97	9.08	2.65 (1.41–4.98)^**^	1.66	26.96	5.27 (2.82–9.88)^***^
Worried about family members to be infected	Yes	0.55	3.15	1.74 (0.94–3.20)	0.82	6.82	2.26 (1.23–4.17)^**^
Someone like friends or neighbors get infected	Yes	0.65	2.85	1.92 (0.90–4.10)	0.30	0.48	1.35 (0.58–3.13)

*B, regression coefficient; OR, odds ratio; CI, confidence interval. Reference categories: age = 18–30 years, sex = male, years of schooling = >10 years, occupation = student/housewives/other, household income affected by COVID-19 = no, Crowding index = normal, shared facilities = 2 or more, food availability for the household = no difficulties, food purchase = more bought/same as before, any food aid received = yes, afraid of being infected by COVID-19 = no, worried about family members to be infected = no, someone like friends or neighbors get infected = no*.

* *p < 0.05*,

** *p < 0.01, and*

*** *p < 0.001*.

Participants with mild insomnia were significantly more likely to share fewer household facilities (OR: 2.32; 95% CI: 1.46–3.70, *p* < 0.01) and to be afraid of being infected by COVID-19 (OR: 3.47; 95% CI: 1.96–6.15, *p* < 0.01; Table 5). Likewise, participants with moderate/severe insomnia were significantly more likely to be female (OR: 1.90; 95% CI: 1.02–3.56, *p* < 0.05), to receive food aid (OR: 0.50; 95% CI: 0.29–0.88, *p* < 0.05), to be afraid of being infected by COVID-19 (OR: 3.85; 95% CI: 1.81–8.19, *p* < 0.01), and to be worried about someone like friends or neighbors getting infected (OR: 2.45; 95% CI: 1.07–5.58, *p* < 0.05).

**Table 5:** Multinomial logistic regression differentiating minimal insomnia (*n* = 335) from mild and moderate/severe insomnia.

**Variable**	**Category**	**Mild insomnia (*****n*** **= 177)**			**Moderate/severe insomnia (*****n*** **= 74)**		
		***B***	**Wald χ^2^ statistic**	**OR (95% CI)**	***B***	**Wald χ^2^ statistic**	**OR (95% CI)**
Age	31–50 years	0.20	0.82	1.22 (0.79–1.89)	0.27	0.79	1.31 (0.72–2.38)
	>50 years	0.07	0.03	1.07 (0.51–2.24)	−0.16	0.08	0.85 (0.28–2.64)
Sex	Female	0.18	0.60	1.20 (0.76–1.90)	0.64	4.04	1.90 (1.02–3.56)^*^
Years of schooling	<5 years	0.16	0.11	1.17 (0.46–3.02)	0.36	0.31	1.44 (0.40–5.17)
	5–10 years	0.56	1.48	1.75 (0.71–4.31)	0.26	0.17	1.29 (0.38–4.45)
Occupation	Unemployed/day laborer	0.20	0.57	1.23 (0.72–2.08)	0.20	0.31	1.23 (0.59–2.53)
	Service	0.12	0.12	1.13 (0.58–2.19)	0.71	2.71	2.04 (0.87–4.75)
	Business/self-employed/garments worker	−0.16	0.27	0.85 (0.46–1.58)	−0.12	0.06	0.89 (0.36–2.18)
Household income affected by COVID-19	Yes	0.42	0.75	1.52 (0.59–3.90)	1.23	2.29	3.41 (0.70–16.68)
Crowding index	Overcrowded	0.19	0.62	1.21 (0.75–1.95)	0.20	0.33	1.22 (0.63–2.36)
Shared facilities	0 to 1	0.84	12.53	2.32 (1.46–3.70)^**^	0.66	3.73	1.93 (0.99–3.74)
Food availability for the household	Difficulties	0.22	0.36	1.25 (0.61–2.56)	−0.04	0.01	0.96 (0.35–2.63)
Food purchase	Bought less	0.63	2.78	1.87 (0.90–3.90)	0.58	1.09	1.79 (0.60–5.34)
Any food aid received	No	−0.07	0.10	0.94 (0.63–1.39)	−0.69	5.80	0.50 (0.29–0.88)^*^
Afraid of being infected by COVID-19	Yes	1.24	18.10	3.47 (1.96–6.15)^**^	1.35	12.20	3.85 (1.81–8.19)^**^
Worried about family members to be infected	Yes	0.19	0.42	1.21 (0.69–2.12)	0.34	0.77	1.40 (0.66–2.96)
Someone like friends or neighbors get infected	Yes	−0.17	0.19	0.84 (0.39–1.84)	0.89	4.51	2.45 (1.07–5.58)^*^

*B, regression coefficient; OR, odds ratio; CI, confidence interval. Reference categories: age = 18–30 years, sex = male, years of schooling = >10 years, occupation = student/housewives/other, household income affected by COVID-19 = no, Crowding index = normal, shared facilities = 2 or more, food availability for the household = no difficulties, food purchase = more bought/same as before, any food aid received = yes, afraid of being infected by COVID-19 = no, worried about family members to be infected = no, someone like friends or neighbors get infected = no*.

* *p < 0.05*,

** *p < 0.001*.

## Discussion

Mental health disorders contribute significantly to the global burden of diseases in LMICs including Bangladesh. Previously researchers found that the mental health of the urban poor was worse than that of the rural poor (31, 32). To date, the impact of the current pandemic on mental health has been studied among young adults and rural women, but the residents of urban slums have been understudied in Bangladesh (31). Our study was the first to examine the impact of the COVID-19 pandemic on mental health (anxiety and insomnia) among people living in urban slums of Bangladesh. The prevalence of mild to severe anxiety and insomnia was 53 and 43%, respectively. Being over 50 years of age and worrying about contracting COVID-19 was significantly associated with risk of mild anxiety. Having less shared facilities, difficulty in availing food, and worries related to COVID infection among self or family members were significantly associated with risk of moderate or severe anxiety. For insomnia, sharing of household facilities was negatively associated and worry about infection was positively associated with mild insomnia. Being female, receiving food aid, and worrying about infection were significantly associated with moderate or severe insomnia.

During this pandemic, there seem to have been a rise in the rates of general mental health conditions such as anxiety and insomnia among the residents of slums in Dhaka city. In studies prior to the COVID-19 pandemic, researchers reported rates of anxiety in urban slum dwellers in Bangladesh to be between 10 and 38% and insomnia to be 18% (33–39). However, in this current study, the rates of anxiety and insomnia were 53 and 43%, respectively, indicating a general decline in mental health status among the urban poor. A similar rise in anxiety disorders was reported among urban poor in India, China, and UK during the COVID 19 pandemic (40, 41). None of the studies described prevalence of insomnia. In addition to individual conditions, about 34.5% respondents presented with both conditions, which can be even more detrimental to the over wellbeing, making them vulnerable to their family members and societies (42). Also, it is important to note that researchers have reported the bidirectional relationship between insomnia and anxiety where anxiety could induce insomnia and vice versa (43, 44).

With regard to age, our study findings showed that people with higher age groups (≥50 years) have a significantly greater risk of anxiety than younger people. Prior to the pandemic, evidence from Bangladesh and India also documented that older people are disproportionately affected by mental illness due to the underlying long-term chronic illness, dependency on their family members, loneliness, etc. (45, 46). Several studies conducted during the COVID-19 pandemic reported that government-imposed isolation causes decrease in wellbeing, lack of social activities, and increase in loneliness leading to have severe impact on mental health particularly among older people (47–50).

In this current study, insomnia was found significantly higher among females compared to males during this pandemic. The global prevalence of mental illness is also found to be higher among women (24.5%) compared to men (16.3%) (51, 52). Several studies conducted during this pandemic also reported the increased rate of mental health symptoms such as anxiety and insomnia among women (53–55). A number of biological, hormonal, social, and cultural factors contribute to this gender disparity in terms of mental illness (56–58).

The significant association between increased number of shared facilities with decreased anxiety and insomnia is important to note. Although during the COVID-19 pandemic the public health officials have discouraged social interaction, our evidence suggests that sharing household facilities with other families, which is probably associated with interacting and socializing with neighbors, was beneficial for mental health as reported by researchers from India as well (59). This finding indicates that for a holistic approach to health, it is important that infection control consideration is coupled with mental wellbeing.

Among our respondents during the COVID-19 pandemic, stressors like difficulties with food availability were significantly associated with increased anxiety. Lack of availability and accessibility of food can lead to food insecurity which is a stressful experience that can result in frustration, feeling of hopelessness, alienation, and feelings of shame that may trigger new or amplify preexisting psychosocial stress (31). In previous studies, researchers have demonstrated that pandemics negatively affect food security of the poor in South Asian countries including Bangladesh (31). In the context of COVID-19, the feeling of psychosocial issues could have been compounded by the imposed social isolation and worries about new health risks (35, 60).

According to our study, participants who received food aid were significantly associated with insomnia. Over the past few years, different support programs are in place to support the residents of the informal settlements including food aids by different organizations (45, 61–64). During the initial outbreak of COVID-19, there were increased numbers of unemployment and lack of food availabilities causing most of the slum residents (31) to rely on food aids by different sources (45, 62, 63). Furthermore, in many instances during the pandemic there was an uneven, demeaning manner of food distribution, unfair political practices, and lack of proper management, documentation, and monitoring which may have rendered many residents to suffer from mental health issues such as insomnia (18, 60, 65–75).

Respondents who were afraid of contracting COVID-19 were more likely to suffer from anxiety and insomnia. Similarly, the respondents who were worried about someone with a close network getting infected had greater odds of insomnia. These findings were also in line with other Western and South Asian countries (24, 76–80). It can be assumed that fear of closed ones being infected, COVID-19-related myths, stigma, and distrust in healthcare bodies have given rise to fear and thus worsened the mental health status of the urban poor (81–83). It is important that we study how to communicate about risk management awareness measures without creating undue anxiety in the future.

Although unprecedented in scale, the experience of the COVID-19 pandemic has demonstrated the gaps in the existing social safety nets for the urban poor. In Dhaka city, 4 million of the urban population live in 5,000 slums and they are a very important part of different sectors such as RMG (readymade garments), transportation, construction, domestic workforce, and part of other local businesses (72, 73). They are a major contributor of the national economy of Bangladesh (84). In the longer term, it is crucial to plan to ensure how this population can be engaged in alternative productive employment opportunities so that they can themselves play a role in the recovery process of the consequences of the emergencies.

### Limitations and Strengths of the Study

There were a few limitations of our study. The study was cross-sectional which means the findings presented were applicable to that point in time only and did not provide us with the insights over time. As the study relied on self-report, this might have resulted in some under- or overestimation due to recall bias or social desirability bias. Due to the restriction of the pandemic, the data were collected over phone which might cause some bias in the assessment of the mental health conditions of the respondents. Slum dwellers were not interviewed who did not have mobile phones or who could not be reached, which may lead to a kind of selection bias. Further, we do not have pre–post data of the same population to assess the additional contribution of COVID-19 pandemic in terms of mental health outcomes.

This study used the PPS methodology, which meant that the sample represents the residents of the five major informal settlements of Dhaka and Gazipur districts. We applied standard validated scales to assess the anxiety disorder and insomnia among the respondents. Furthermore, we have collected all the information just after the initial lockdown was over, which means that the findings represent the effect of the lockdown on mental health.

## Conclusions

The findings indicate that anxiety and insomnia were prevalent and higher than previously observed. This study emphasizes the need to integrate the consideration of mental wellbeing in the infection control mechanism for the present pandemic as well as for similar situations in the future. Our study also recommends that during emergencies such as one presented by COVID-19, it is important that the health and wellbeing of the urban poor are safeguarded through a well-designed comprehensive social safety net program. Special effort could be made to educate communities about how to be safe while having social interactions. Primary care facilities could be equipped to deal with identification and referral of mental health conditions, and local opinion leaders could be engaged in talking about the importance of mental health. Finally, in the context of severe shortage of trained staff who are equipped to deal with mental health conditions, it is important that telemedicine and other online counseling facilities are made accessible and affordable for the urban poor so that their wellbeing can be ensured.

## Data Availability Statement

The raw data supporting the conclusions of this article will be made available by the authors, without undue reservation.

## Ethics Statement

The studies involving human participants were reviewed and approved by the Institutional Review Board (IRB) of icddr,b [PR-20075]. The participants provided their verbal consent through phone calls to participate in this study.

## Author Contributions

KK and SR: conceptualization. KK, SR, FK, SM, SH, and DR: methodology. MI, KK, MK, and SR: formal analysis and investigation. KK, MK, and MI: writing—original draft preparation. SM, SH, DR, FK, and SR: writing—review and editing. SR and DR: resources. SR: supervision. All authors have read and approved the final manuscript.

## Funding

This study was funded by the Swedish International Development Cooperation Agency (SIDA), and the Grant Number was GR-01455. No other specific grant from any funding agency or from any other non-profit sectors was received for this research.

## Conflict of Interest

The authors declare that the research was conducted in the absence of any commercial or financial relationships that could be construed as a potential conflict of interest.

## Publisher's Note

All claims expressed in this article are solely those of the authors and do not necessarily represent those of their affiliated organizations, or those of the publisher, the editors and the reviewers. Any product that may be evaluated in this article, or claim that may be made by its manufacturer, is not guaranteed or endorsed by the publisher.
